# Geographic and Opportunistic Recovery with Depth and Power Transmission Adjustment for Energy-Efficiency and Void Hole Alleviation in UWSNs

**DOI:** 10.3390/s19030709

**Published:** 2019-02-09

**Authors:** Abdul Mateen, Muhammad Awais, Nadeem Javaid, Farruh Ishmanov, Muhammad Khalil Afzal, Saqib Kazmi

**Affiliations:** 1 Department of Computer Science, COMSATS University Islamabad, Islamabad 44000, Pakistan; ammateen49@gmail.com (A.M.); amawais@hotmail.com (M.A.); saqib.kazmi149@gmail.com (S.K.); 2 Department of Electronics and Communication Engineering, Kwangwoon University, Seoul 01897, Korea; 3 Wah Campus, COMSATS University Islamabad, Wah Cantonment 47040, Pakistan; khalilafzal@ciitwah.edu.pk

**Keywords:** GEDPAR, void holes, energy efficiency, Underwater Wireless Sensor Networks (UWSNs), depth adjustment, transmission range

## Abstract

Underwater Wireless Sensor Networks (UWSNs) are promising and emerging frameworks having a wide range of applications. The underwater sensor deployment is beneficial; however, some factors limit the performance of the network, i.e., less reliability, high end-to-end delay and maximum energy dissipation. The provisioning of the aforementioned factors has become a challenging task for the research community. In UWSNs, battery consumption is inevitable and has a direct impact on the performance of the network. Most of the time energy dissipates due to the creation of void holes and imbalanced network deployment. In this work, two routing protocols are proposed to avoid the void hole and extra energy dissipation problems which, due to which lifespan of the network increases. To show the efficacy of the proposed routing schemes, they are compared with the state of the art protocols. Simulation results show that the proposed schemes outperform the counterparts.

## 1. Introduction

The planet Earth, on which we live our lives, consists of 70% water. Whereas the oceans hold more than 90% of total water. This much quantity shows the importance of the water medium. To explore the underwater medium for getting and sharing important information, a network is deployed in a specific region. Information transmission using Underwater Wireless Sensor Networks (UWSNs) is one of the emerging technologies and is used for the betterment of ocean observation systems. Applications of UWSNs range from aquaculture to oil industry; instrument monitoring to climate recording; pollution control to predictions on natural disasters; search and survey purposes to submarine purposes.

The sensor node in an UWSN acquires the desired information and transmit towards the next forwarder node which is closer to the sink (Sink: This word is alternatively used as sink node, sonobuoy, destined node and destination node) [1]. This sink may be the onshore data center or a simple sensor node over the water surface. In data forwarding procedure, the source (Source: The words source node and initial node are alternatively used for the source) node generates data packets and communicate with its neighbors to find the potential node. Afterward, the potential neighbor node finds the next potential node from its neighbors and transmits data packets towards that potential node. To find the potential neighbor from the forwarder node, some criteria and routing procedures are defined. This criterion may base upon efficient energy use or alleviation of void holes.

Radio Frequency (RF) waves cannot be used in an underwater medium. The reason behind is that RF technology increases the energy consumption by increasing the attenuation factor. At lower frequencies (30 to 300 HZ), water becomes a conductor for RF waves. Moreover, the frequencies in this range require the large size of antennas and these antennas require high transmitting power for data transmission. These requirements cannot be fulfilled in UWSNs. Consequently, RF waves cannot be used in UWSNs. Additionally, the technology of optical waves requires very high precision on a single point for a transmitter and a receiver. However, sensor nodes move with the water current. In essence, we have to use the acoustic waves in UWSNs. Nevertheless, the speed of acoustic waves is almost five orders of magnitude less than the speed of RF waves [2].

The underwater medium is extremely unpredictable and challenging when compared with Wireless Sensor Network (WSN). The major differences of UWSN with WSN are: (1) high energy consumption (2) high propagation delay (3) low bandwidth (4) high dynamic topology-operation (5) less propagation speed (6) low efficiency (7) low data transmission rate and (8) high environmental and noise interferences. The comparison between UWSN and WSN is shown in Table 1.

The design of routing protocols has paramount importance in UWSN. These protocols indicate the routing path for data from the source node at the bottom towards the sinks node at the surface of the ocean. Expressly, these protocols face the different challenges which are associated with the underwater medium, e.g., limited battery resources, interference, noise, reliable Packet Delivery Ratio (PDR), high propagation delay, movements of sensors and void holes.

Efficient energy usage is one of the most important tasks during the design of a routing protocol. As the batteries of sensor nodes in an underwater environment are non-removable and have limited energy storage. This issue provides a strong base for efficient battery use. Mostly, energy dissipates during the processes of data packet transmission and reception. Efficient energy usage depends on various factors. For instance, the initial position and number of anchor nodes; sensor nodes and the way in which nodes are deployed. The deployment of a network must be one of the two types (1) sparse deployment and (2) dense deployment. The sparse deployment leads toward the creation of a void hole and dense deployment results in an excessive amount of sensors failure.

The energy and network stability have a direct relation. As the energy of sensors increases, the stability of the network will be longer, and vice versa. Void holes are areas within the transmission range of a network where a node cannot find its next neighbor or forwarder. The void holes creation has following reasons (1) node becomes dead due to a lot of energy usage and (2) no forwarder node.

Topology control has the ability to overcome the undesired effects of UWSNs and consequently to enhance the performance of routing protocols. The relationship between topology control and UWSN is summarized as follows [3]

- In UWSNs, wireless communication is provided by the acoustic channel to enable networking services.
- UWSNs have many peculiar characteristics that enhance the challenges in effective networking design.
- To overcome these challenges and increase the performance of the network, the topology control method is the best solution.

Localization of sensor network in underwater is indispensable. The gathered data is useless until it is not correlated with the specified position of the sensor node. Localization in UWSNs is very important as it has many useful applications, e.g., target tracking, underwater environment monitoring, pollution control and geographic routing protocols. Nevertheless, UWSNs cannot use the Global Positioning System (GPS) due to high energy dissipation and high attenuation of RF signals [4,5].

In this work, we proposed GEographic and opportunistic routing with Depth and Power Adjustment Routing (GEDPAR) and End to End Void Hole Recovery (E2EVHR) routing techniques. GEDPAR and E2EVHR are compared with GEographic and opportunistic routing with Depth Adjustment Routing (GEDAR) and Layered Multi-path Power Control (LMPC) state of the art routing protocols. Simulations are performed to check the effectiveness of the proposed schemes.

The remainder of this work is organized as follows: Section 2 provides a brief overview of state of the art work. The problem statement is elaborated in Section 3. Section 4 presents the proposed system model. Discussion on the simulations is given in Section 5. Feasible regions of the proposed protocols are presented in Section 6. Finally, Section 7 summarizes the whole work.

## 2. Related Work

In this section, we review and compare some recent works on the basis of covering a specific area of UWSNs. The works which cover the energy efficiency and void holes are compared in Section 2.1. Additionally, the works that cover the concept of localization or geographic routing are compared in Section 2.2. Moreover, Section 2.3 presents the comparison of topological control based schemes. Finally, the concept of a void hole is presented in Section 2.4. Moreover, Table 2 provides the summary of these works.

### 2.1. Energy Efficiency Based

The works [6,7,8,9] proposed different schemes to enhance the energy-efficiency. The works in [6,7] have used the multi-hop techniques. Whereas, the paper [6] is focusing on network reliability, mobility management, PDR and energy efficiency. On the other hand, the work [7] is only focusing on energy efficiency. Both works [6,7] achieve their objectives; however, end-to-end delay is compromised. The authors in works [8,9] mainly focus on reliability by covering one-hop from the forwarder node. The proposed scheme EBLE from the work [8] aims to minimize the energy dissipation with packet size management. The objective is successfully achieved on the cost of delay. The cooperative routing is used in work [9] for data reliability and mobility management, while PDR and efficient energy usage are the main aims. The objectives are achieved successfully; however, the network performs poorly in sparse network deployment.

The works [10,11,12,13] are also using energy efficiency techniques. The works [10,12] provide the reliability. Both of works discuss the concept of multi-hoping. The proposed scheme in the work [10] is beneficial for a large amount of data packets; however, this proposed technique does not perform well in sparse network deployment. The MLPR from [12] looks toward the efficient path for routing by using minimum energy. For the implementation of MLPR, more memory is required for the extra operations at each node. The energy dissipation schemes; SDVF and EBULC are proposed in works [11,13], respectively. Both schemes consider mobility management for decreasing the energy consumption in UWSNs. Results show that end-to-end delay in the works [11,13] is enhanced.

The energy efficiency is focused in the works [14,15,16,17]. In [14], some data collection methods are discussed which used minimum energy for data transmission from source to the destination. In both [14,15], mobility management is considered, while in the [14], reliability and packet size management is not considered. Nevertheless, the papers [15,16,17] focus on the reliability of the network. Additionally, [15] considers both types of forwarding strategies; single-hop and multi-hop. While [14,16,17] only focus on single-hop from the current node. Moreover, the work in [15] considers the security issues of UWSNs. While in [14], the authors discusses the problems of getting route information. In [16], the complexity of the network is a major challenge. Additionally, paper [17] works for energy efficiency by managing the size of data packet.

### 2.2. Localization Based

The authors in [1,2,4,18,19] discuss the geographic or localization-based routing. The work in [1,2] review the works in which the concept of localization-based routing is used. Both of these above, discuss reliability and none of them work on mobility management or packet size management. Moreover, in [1,2], the concept of single-hop and multi-hop is devised. The challenges which are discussed in these works are high interference, limited batteries of sensor nodes, low bandwidth and malicious attacks. The work in [18] achieves the higher PDR by finding the locations of alive nodes. Afterward, the data packets are sent to these alive nodes, accordingly. The challenges discussed in [19] are localization, feasible hardware, relevant simulation tools and low power gliders.

### 2.3. Topology Control Based

The authors in [3,5,7,20] proposed topology control-based solutions. TCEB and GARM schemes are proposed for controlling the topology of UWSNs in [7,20], respectively. In addition, the [3] classifies different topological protocols. From [3], reliability and mobility is discussed. The work [3] focuses on single-hop and multi-hop while the work [7] only focus on next forwarder node. The challenges that discussed in [3,5,7,20] are: high attenuation, mobility of sensor nodes, energy efficiency, low bandwidth, connectivity loss, high bit rate error, high deployment cost, complexities and optimal location of glider. Using dynamic topological strategy, work in [7] achieves energy efficiency and the work in [20] enhances both PDR and energy efficiency. In [5], mobility management is a major consideration using EEL and the concept of multi-hoping. In addition, the work [5] achieves better simulation results from compared ones.

### 2.4. Void Hole Based

The concept of a void hole is presented in [21,22,23,24,25,26]. Void holes are the regions within the network range from where further data delivery is not possible. In other words, if a forwarder node does not have any further node for data packet transmission then this node is called void node and the area where transmission is not possible in called void holes. TORA is presented in [21] in order to avoid the void holes. The proposed scheme uses the concept of multi-hoping to avoid void holes and to improve energy efficiency. Nevertheless, reliability and complexity of this scheme are not discussed.

## 3. Problem Statement

In UWSN, each sensor has limited resources and requires effective use of these resources. Efficient energy consumption has a major contribution to stabilizing the network for long-term communication. In UWSNs, the packet is sent from the source node to the sink node using different relay nodes. If a node cannot find a forwarder node in its transmission range, it causes hindrance in the network during communication.

To avoid the void holes in UWSNs, a routing protocol namely GEDAR presented in [22]. GEDAR addresses the issue by adjusting the depth of nodes; however, the process of depth adjustment consumes lots of energy. In [23], LMPC routing technique addresses the efficient data transmission by making the binary tree from the root node. However, binary tree generation consumes high energy and lead towards the transmission overhead. To overcome the aforementioned problems, two routing protocol namely GEDPAR and E2EVHR are proposed for avoiding the void holes and eliminating the extra energy consumption.

## 4. System Model

**Figure 1:**
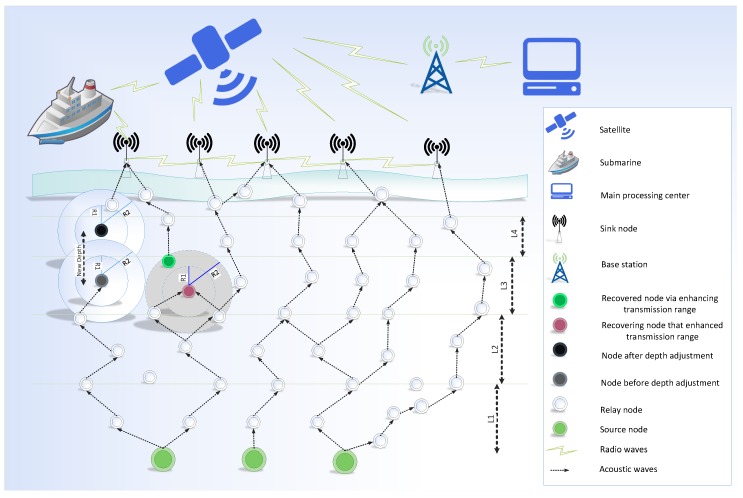
Proposed system model.

In this section, our proposed system model is presented in Figure 1. The system model consists of source nodes, relay nodes and sonobuoys. Source node forwards data packets toward the destined sonobuoys during transmission. The proposed protocol follows multi-hoping feature for packets transmission. Source and relay nodes only use acoustic signals while radio waves are used for communication among sink node, submarine, satellite, base station and the main processing unit.

In the proposed system model, sensor nodes are randomly deployed in an underwater medium. Nevertheless, sink nodes are deployed at the sea surface. The same transmission range and energy are assigned to each sensor node. Moreover, each sensor node has also the ability to adjust their depth from the lower layer to the upper layer. During depth adjustment, nodes only move in a vertical direction. The process of depth adjustment occurs in the case when a node cannot find its next forwarder even by increasing the transmission range. There are three different cases that are elaborated through the proposed system model.

- Successful transmission
- Occurrence of a void hole
- Void hole recovery

**Successful Transmission**

In the current scenario, successful transmission occurs when a packet which is generated from the source is successfully received at the sink node. In this case, if a packet is transmitted from the initial node, it follows different paths to reach a destination. During transmission process, packet moves from one depth to other and one layer to other. However, the direction of the packets is the sink node.

**Void Hole Occurrence**

**Figure 2:**
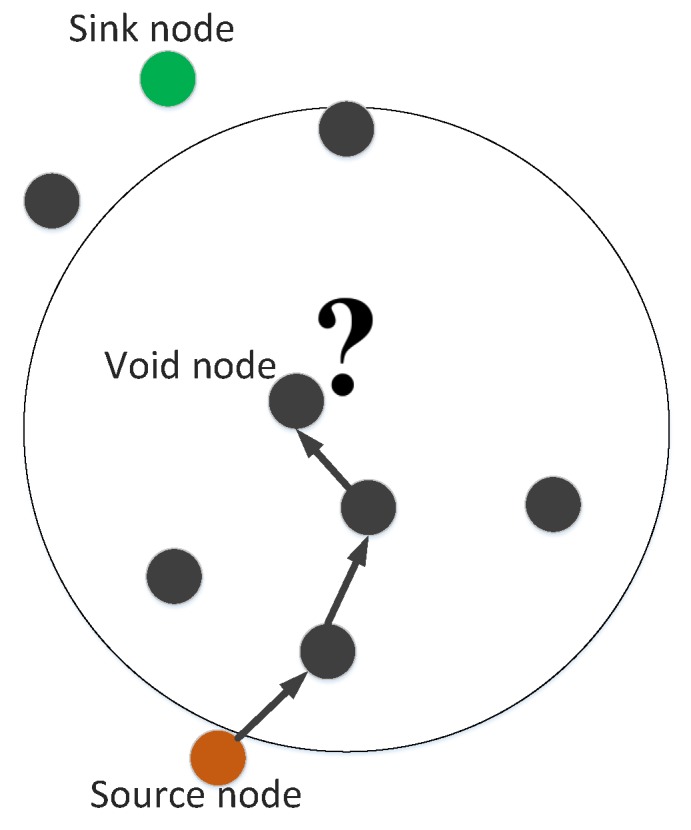
Void node.

Void holes can be defined as nodes having no neighbors in their communication range. Void holes are generated due to many reasons; e.g., (1) A node can be dead due to again and again re-selection of the same node or (2) No next forwarder node exists in the transmission range of current forwarder node. The occurrence of void hole and blockage in transmission is summarized in Figure 2.

**Void Hole Recovery**

On the occurrence of void holes, we have to perform some recovery methods. In this work, GEDPAR is proposed for void hole recovery purpose. GEDAR and LMPC are also used for the same purpose. However, both of these methods have some limitations, e.g., GEDAR always takes depth adjustment on the occurrence of a void hole. On the other hand, LMPC uses the concept of the binary tree and forward multiple copies of each packet.

**Figure 3:**
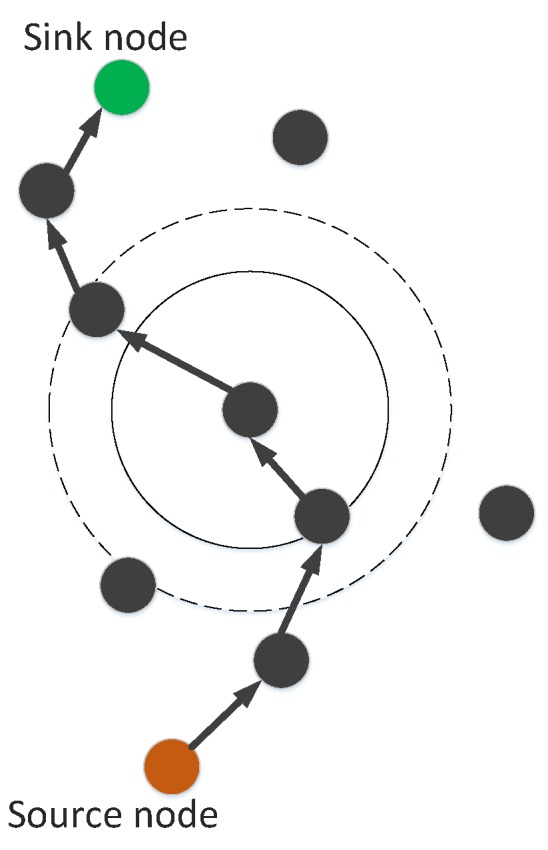
Transmission range adjustment.

**Figure 4:**
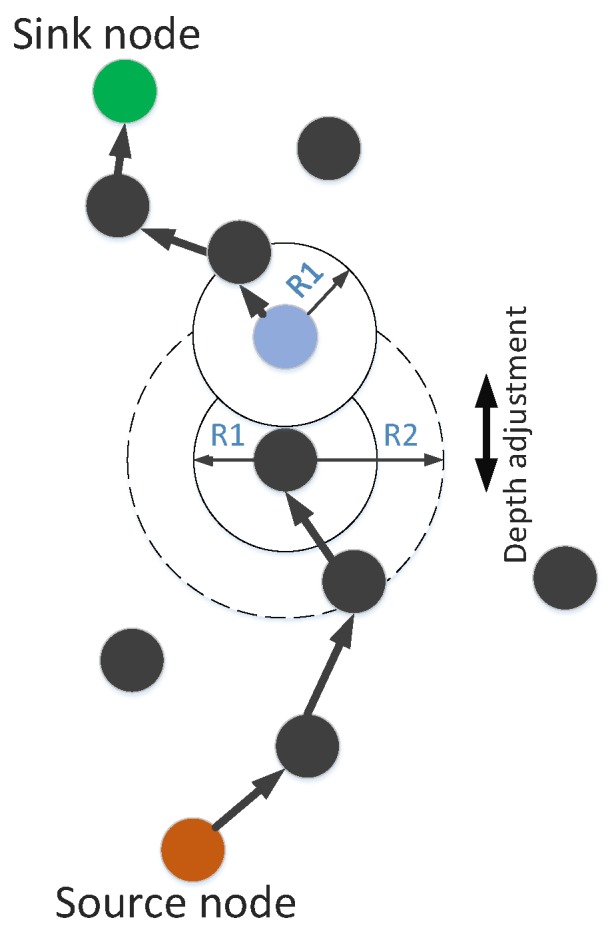
Depth adjustment.

In the proposed scheme, we can use the option of an increase in transmission range or depth adjustment. Depth adjustment is used in the case when a neighbor node could not found even in maximum transmission range. Figure 3 presents the transmission range adjustment. The solid circle shows the original transmission range while dotted circle shows the enhanced transmission range. Depth adjustment procedure is shown in Figure 4. In this figure, R1 shows the minimum transmission range and R2 indicates the maximum transmission range. In case, if a node cannot find next forwarder in R2 then the current node must adjust its depth vertically. The node in blue color shows the new depth.

### 4.1. Establishing the Link with Neighbors

**Figure 5:**
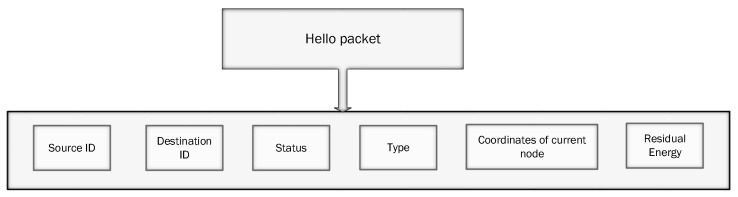
Structure of hello message.

Sensor nodes are deployed in an underwater environment and each sensor generates a hello message (control message) to find its neighbors. This hello message is a tuple of several things: source-id, destination-id, the status of a node (dead or alive), type of node (source node or sink node), coordinates node and residual energy of current node. This structure is presented in Figure 5.

The estimated and the actual distances of each neighbor are calculated on the basis of coordinates.

### 4.2. Forwarder Node Selection

According to our proposed system model, neighbor node is selected on the optimality basis. The criteria for optimality in this scenario for a node is to have efficient energy and must lead towards the sink node. Optimal energy point for a node is calculated using the following formula:

(1) $$DistSD_{op}=\sigma \sqrt{\frac{B}{\left(1-2^{\left(1-\sigma \right)}\right)}}.$$

Here, $DistSD_{op}$ represents the optimal distance from source to the destination node. Where, σ is a path loss constant and calculation for *B* is as follows:

(2) $$B=2\times E_{com}.$$

where, $E_{com}$ represents the communication energy. The slope for straight line *m* from source node to the destination node is calculated as:

(3) $$m=\frac{y_{S}-y_{D}}{x_{S}-x_{D}}.$$

Here, $y_{S}$, $y_{D}$, $x_{S}$ and $x_{D}$ are the coordinates of sending and receiving nodes. Equation (4) describes the path loss during attenuation of signal. This equation is taken from [22].

(4) $$A\left(d,f\right)=Dist{SD_{op}}^{k}a{\left(f\right)}^{DistSD_{op}},$$

where, a(f) is the absorption coefficient, *k* is a spreading factor and $DistSD_{op}$ indicates the optimal distance from a source to the destination node. The absorption coefficient is described by Throp’s formula.

(5) $$10loga\left(f\right)=\left(\frac{0.11\times f^{2}}{1+f^{2}}\right)+\left(\frac{44\times f^{2}}{4100+f^{2}}\right)+2.75\times 10^{-4}f^{2}+0.003.$$

Transmitting probability for any node with distance d is calculated using Equation (6).

(6) $$P\left(d,m\right)={\left(1-P\left(DistSD_{op}\right)\right)}^{m}.$$

Turbulence noise $Noise_{tur}$, shipping noise $Noise_{ship}$, wind noise $Noise_{wind}$ and thermal noise $Noise_{ther}$ are calculated using Equations (7)–(10), respectively.

(7) $$Noise_{Tur}=10^{\left((17-30\times log10(f))\times 0.1\right)}.$$

(8) $$Noise_{ship}=10^{((40+20\times (shp-0.5)+26\times log10(f)-60\times log10(f+0.03))\times 0.1}.$$

(9) $$Noise_{wind}=10^{\left(\left(50+{\left(\left(7.5\times wind\right)\right)}^{0.5}\right)+\left(20\times log10\left(f\right)\right)-40\times log10\left(f+0.4\right)\right)}.$$

(10) $$Noise_{ther}=-15+\left(20\times log10\left(f\right)\right).$$

Whereas, total noise $Noise_{total}$ is calculated by adding all noises as:

(11) $$Noise_{total}=Noise_{Tur}+Noise_{ship}+Noise_{wind}+Noise_{ther}.$$

### 4.3. GEDAR

GEDAR is an opportunistic and depth adjustment-based routing protocol. In GEDAR, each packet is sent to the forwarding set which consists of several neighbors. Algorithm 1 shows the procedure of periodic beaconing in GEDAR. This procedure requires S and D. Where, κ represents beacon messages. Lines 4–16 elaborate on the overall procedure for distance and neighbor calculations. Lines 8–11 add neighbors to the neighbor list. Line 6 shows that this procedure repeats for each and every source node. Similarly, Algorithm 2 shows the steps upon receiving the beacon message. From Algorithm 2, lines 5–10 show the information update of neighbors, while lines 7–8 show that the sequence of neighbor node is updated.

Algorithm 3 elaborates the selection procedure of next potential node. The packet will only be sent to the potential node and this criterion is defined in Algorithm 3. Where, $\varsigma_{j}$ is a copy of ${∁}_{j}$ and n1 shows the highest priority of node. In this algorithm, firstly, we define the set of candidate neighbor nodes. Each time $\varsigma_{j}$ takes a copy of ${∁}_{j}$ for maintaining the list of nodes within its transmission radius.

Algorithm 4 involves the steps for the recovery of the void hole. First of all, value for the current node is set to “1” for its identification and stops the beacon messages (lines 1–3). The symbol ∅ shows that current node has no neighbor (line 4). In other words, it is a void node. ν is a set which contains the record of next forwarder nodes. Δ and $n_{v}$ are the void nodes set and current void node, respectively. The distance for each forwarder near the current void is calculated in line 10. In lines 11–14, this distance is compared with the transmission range. If the distance is less than the transmission range, then the next forwarder node is within the range of the current forwarder node and vice versa. In case, if no forwarder node exists within transmission range then depth adjustment takes place and the status for the void node is set to “0” from “1” (lines 17–18).

**Algorithm 1** Periodic beaconing

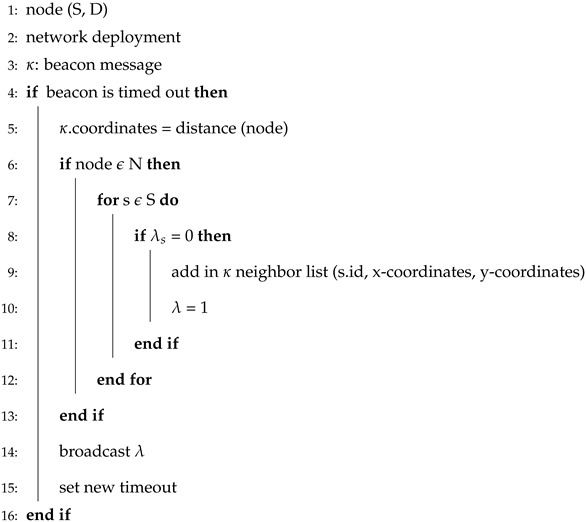



**Algorithm 2** Beacon receive

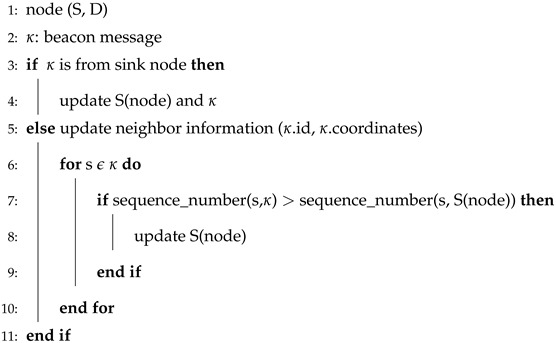



**Algorithm 3** Next forwarder node selection

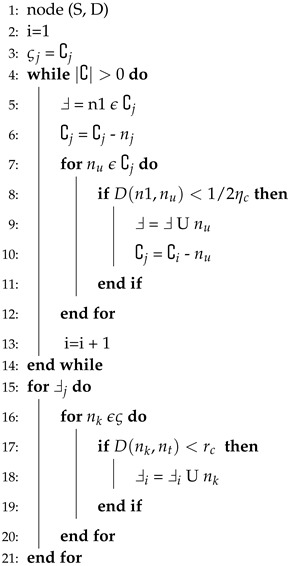



**Algorithm 4** Void hole recovery for GEDAR

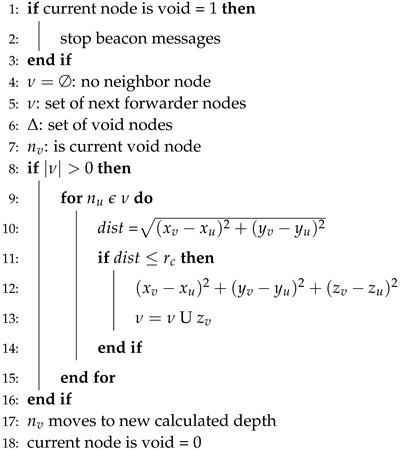



### 4.4. LMPC

**Figure 6:**
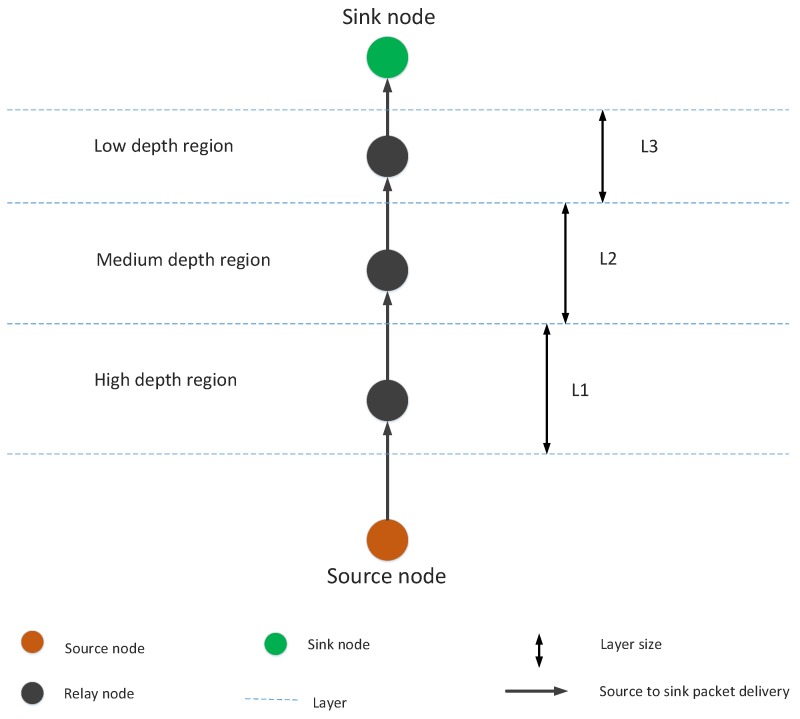
LMPC layer concept.

In LMPC, for efficient transmission, multi-layer concept is implemented by dividing the network environment into unequal layers; as, the working of LMPC totally depends on the layers. We have already discussed that noise in deep water is less than the shallow water. So, the size of a layer in deep water is greater than the shallow water. The size of a layer has an inverse relation with noise attenuation; greater the attenuation of noise, lower will be the layer size and vice versa. The pictorial form of these layers is presented in Figure 6 and Algorithm 5 elaborates the steps for LMPC routing scheme. From Algorithm 5, line 1 shows the input and line 2 represents the parameters’ initialization. Total energy of each node is calculated in lines 3–5. Total number of layers in which network is divided are decided in line 6. Line 7 shows the deployment of nodes. Neighbor finding procedure is done at lines 8–11. The start of the communication is represented by the line 12. On successful communication, acknowledgement message is sent to the source node.

### 4.5. GEDPAR

GEDPAR is our proposed routing protocol. To show the efficacy of the proposed protocol, GEDAR and LMPC are taken as benchmark schemes. In GEDPAR, layering concept is taken from the LMPC and depth adjustment is taken from the GEDAR. GEDPAR takes transmission enhancement step on the appearance of void holes. Transmission enhancement takes some extra energy; however, most of the void holes are removed in this process. If a node cannot cover the void hole even by increasing the transmission range then depth adjustment takes place for that node. Figure 1, Figure 2, Figure 3 and Figure 4 show the pictorial summary of the proposed algorithms. Working of Algorithms 1–3 are the same for GEDAR and GEDPAR. However, Algorithm 6 plays an important role for differentiating GEDPAR from GEDAR.

**Algorithm 5** LMPC

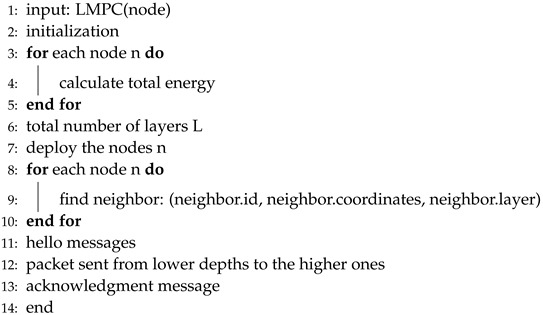



**Algorithm 6** Void hole recovery for GEDPAR

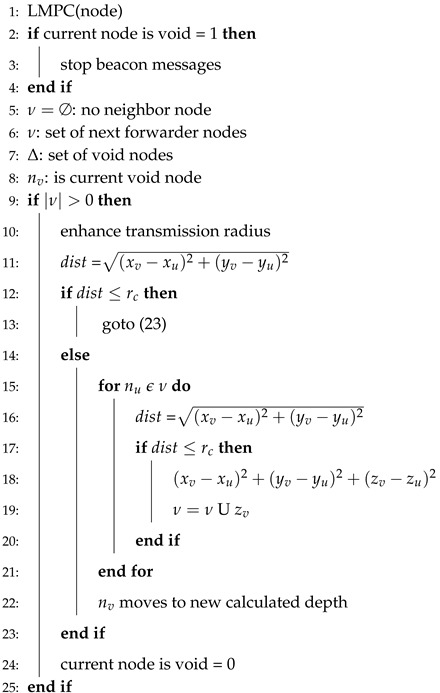



### 4.6. E2EVHR

**Figure 7:**
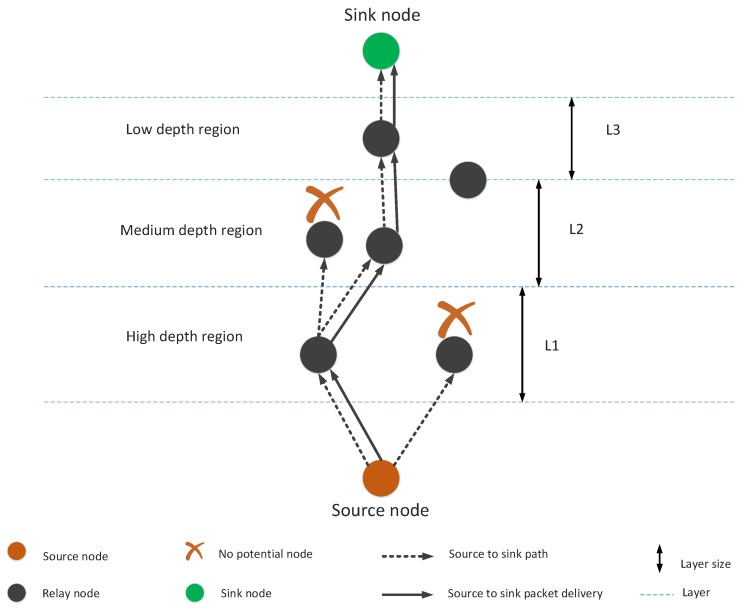
Source to destination path finding.

This proposed protocol is the improved version of LMPC. However, in this routing protocol first of all complete route finding procedure is executed. In E2EVHR, binary tree is generated (as in LMPC) for ensuring the successful packet transmission. Actually, after ensuring that there is no void hole in the routing path, multiple copies of the packets are transmitted towards the sink node using binary tree. The layering concept for LMPC is presented in Figure 6. The major difference between LMPC and E2EVHR is that E2EVHR avoids the void hole by looking the path from source to the destination. (Figure 7), while LMPC looks forward one-hop from neighbor node.

Algorithm 7 presents the steps for E2EVHR protocol. The lines 1–2 are presenting the input and initialization, respectively. The line 3 involves the layering while line 4 shows deployment of the nodes in the network. Total energy for each node is calculated in lines 5–7. The neighbors for each node are calculated in lines 8–10. In lines 12–15, the paths having no void node are evaluated. The lines 16–17 help to find the potential neighbors from the current forwarder. The packets are then transmitted toward the potential neighbor for continuing the process of transmission. These potential neighbors help the packets to reach at the destination, successfully.

**Algorithm 7** E2EVHR

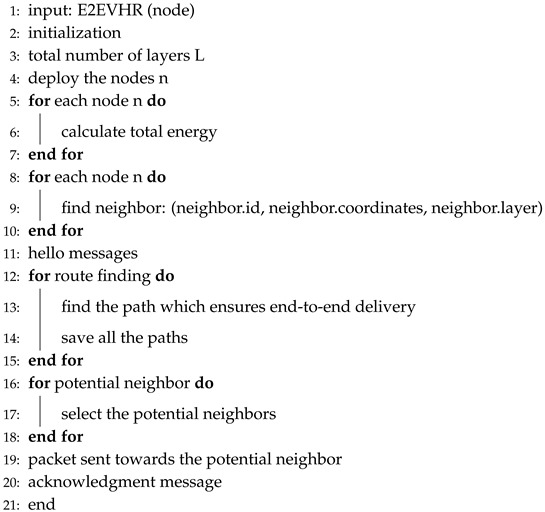



Algorithms are processed according to the following order. Firstly, Algorithm 1 is run which takes the input as sensor node S and sink node D. Secondly, Algorithm 2 is called in the response of Algorithm 1. After that, Algorithm 3 is called for potential neighbor selection. In the end, Algorithm 6 is called (in case of void hole occurrence) for void node recovery. While in the case of E2EVHR, void holes are avoided by finding the path which ensures end to end delivery.

## 5. Simulation and Discussion

Simulations are performed to validate the proposed routing techniques. The proposed techniques are compared with GEDAR and LMPC. GEDPAR and E2EVHR are greedy opportunistic routing protocols in which next forwarder node is selected on the criteria of minimum distance from the current node. In the GEDPAR, firstly, current node enhances transmission range when it finds no neighbor in its transmission range. After that, if the current forwarder still not able to find any node in its range then it executes depth adjustment. During depth adjustment, the node moves from a deeper layer to the shallow one. The second proposed routing algorithm (E2EVHR) takes some steps from LMPC.

Performance of GEDPAR and E2EVHR are compared on the basis of delay, throughput, Packet Acceptance Ratio (PAR), depth adjustment and energy consumption. These parameters can be defined as follows:

### 5.1. Delay

It can be defined as the time duration in which packet is successfully received at a sink. It is measured in milliseconds (msec).

### 5.2. Throughput

Throughput is calculated as the total number of packets received at the sink node to the total number of packets sent from source node in percentage.

### 5.3. PAR

PAR is a ratio between packets received at the sink to the packets sent from the source node.

### 5.4. Depth Adjustment

The number of times nodes move from deep water to the shallow water to find the next neighbor node in their range.

### 5.5. Energy Consumption

Total energy consumption of network during transmission, reception, transmission range enhancement and depth adjustment. This total energy consumption of network is measured in Joule (J).

### 5.6. Network Parameters Setting

The network is deployed over the area of 1500 m × 1500 m × 1500 m. The number of nodes and sinks are 100 and 45, respectively. Initially, nodes are deployed randomly. The initial transmission range of each node is 245 m and nodes can transmit up to 270 m using some extra energy. This happens only when current forwarder cannot find the next node in its transmission area. The initial energy of each node is 100 J. The velocity of acoustic waves and bandwidth for the network is considered 1500 m/s and 3000 kHz, respectively. Transmission energy, reception energy and idle time energy are considered as 2 W, 0.1 W and 10 × $10^{-3}$ W, receptively. Size of hello packet is 100 bytes while the sizes of all other packets are 150bytes. Summary of these parameters is presented in Table 3.

### 5.7. Simulation Results

**Figure 8:**
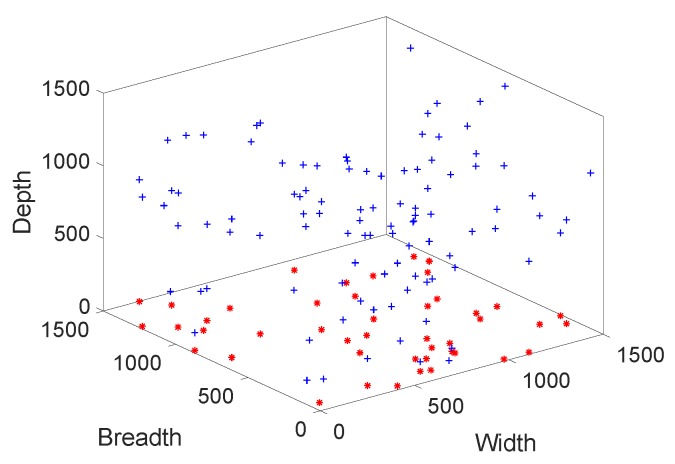
Initial deployment.

Initial deployment of our model is presented in Figure 8. This is a 3D deployment which covers x-axis, y-axis, and z-axis, respectively. The x-axis represents the width of the deployed wireless network. Y-axis indicates the breadth and z-axis cover the depth of the network. From the Figure 8, red “*” and blue “+” are representing the sink nodes and source nodes, respectively.

**Figure 9:**
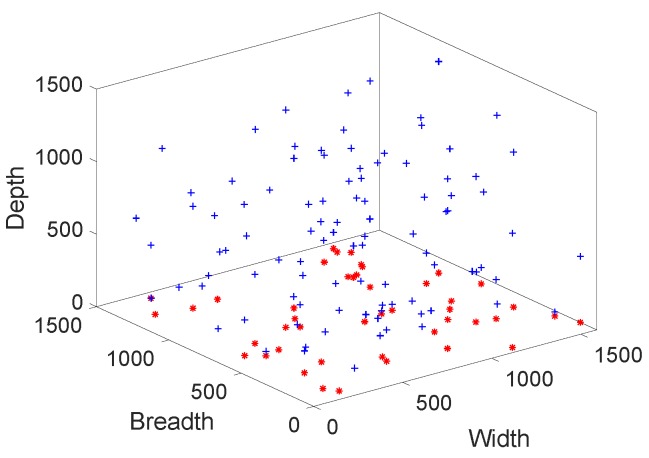
Final deployment.

In Figure 9, final deployment is presented. This final deployment is done after adjusting the transmission range and depth of all nodes. These transmission ranges and depths are only adjusted in case of void hole occurrence. When void hole occurs, first transmission range is enhanced for finding the next forwarder node using some extra energy. If none of the forwarder neighbors is found then depth adjustment occurs. Depth adjustment is done after enhancing transmission range because it consumes more energy.

**Figure 10:**
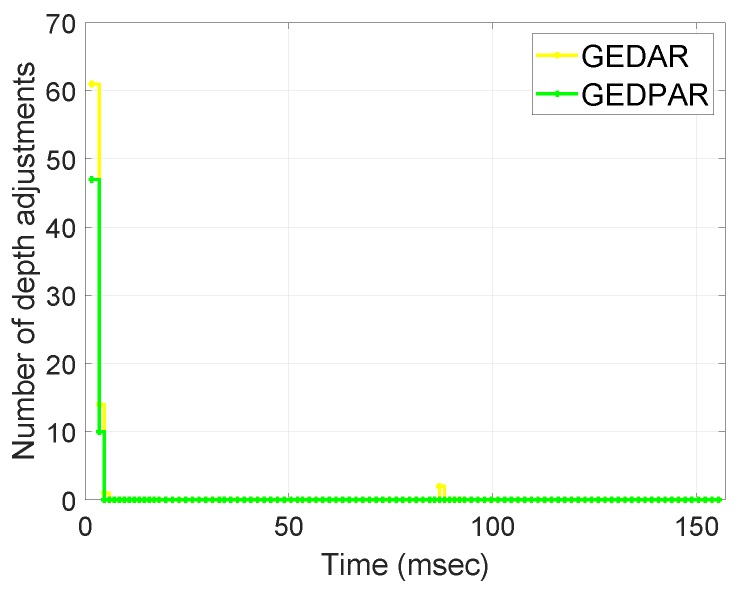
Depth adjustment.

Figure 10 depicts the depth adjustment of nodes. We can see from the Figure 10 that most of the depth adjustment is done during the start of network deployment. Once the network is deployed and initial depth adjustments are done then there exist only a few occasions on which depth adjustment is required. A large amount of energy is dissipated during the process of depth adjustment. So, we make sure that the depth adjustment only occurs when it is necessary. Otherwise, try to avoid the nodes by enhancing the transmission range. It is clear from the Figure 10 that in GEDPAR routing protocol, the nodes require fewer depth adjustments as compare to GEDAR. This step further involved in lessening the energy dissipation.

**Figure 11:**
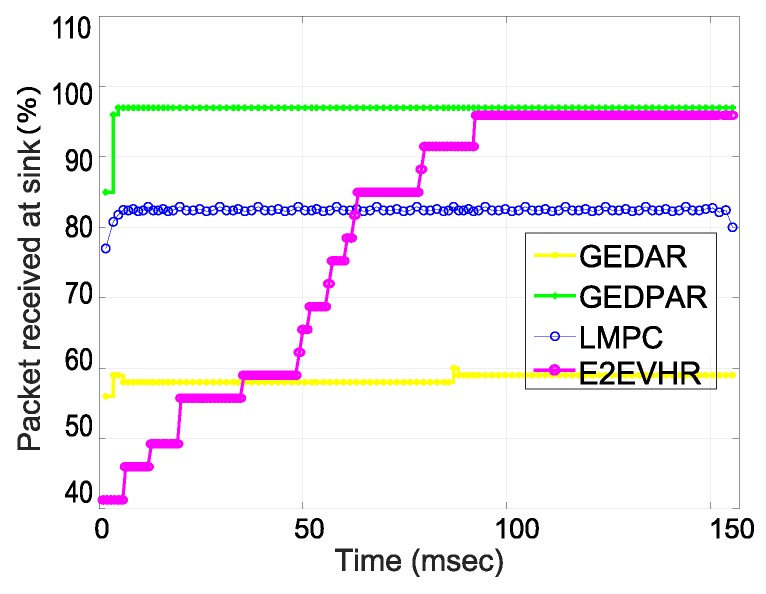
Throughput.

The throughput of proposed routing protocols is compared with GEDAR and LMPC. Figure 11 shows this comparison and assures the efficiency of proposed schemes. According to simulation results, LMPC performs better than GEDAR while GEDPAR outperforms both GEDAR and LMPC. The efficiency of the proposed scheme is better than LMPC and GEDAR by the percentage of 13% and 37%, respectively. As we can see from the Figure 11 that the second proposed technique also outperforms the counterparts. Firstly, E2EVHR performs poor during the end to end node finding process; however, when the proposed scheme finds the end to end routes than its throughput is improved drastically.

**Figure 12:**
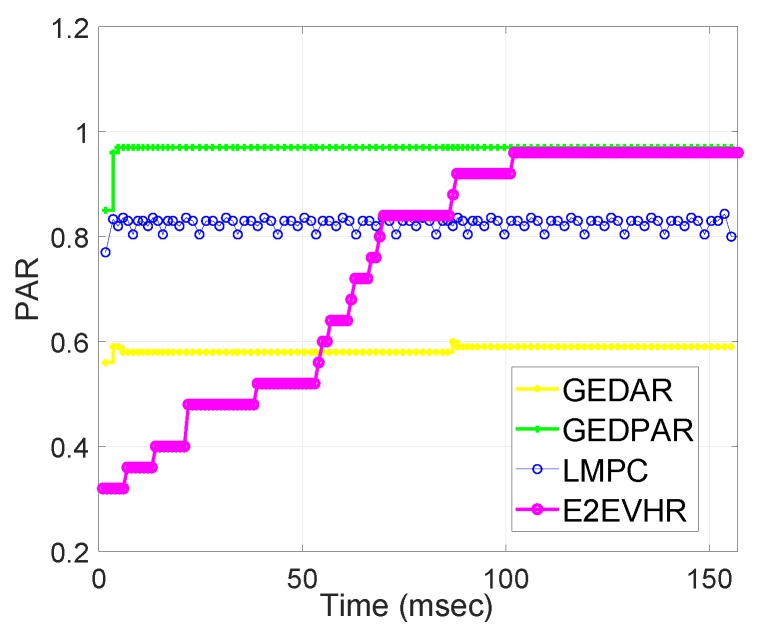
Packets received at sink.

Figure 12 shows the performance of GEDAR, GEDPAR, E2EVHR and LMPC with respect to PAR. PAR is already defined in Section 5.3 as it is a ratio between packets received at sonobuoys to the packets sent from source nodes. We measure PAR from the range of 0 to 1. Here, 0 indicates the minimum PAR and 1 represents maximum PAR. In our scenario, during PAR calculation it is noticed that PAR of GEDAR, LMPC, E2EVHR and GEDPAR is about 0.6, 0.8, 0.93 and 0.95, respectively. These statistics clearly show that GEDPAR and E2EVHR perform best among the comparing ones.

**Figure 13:**
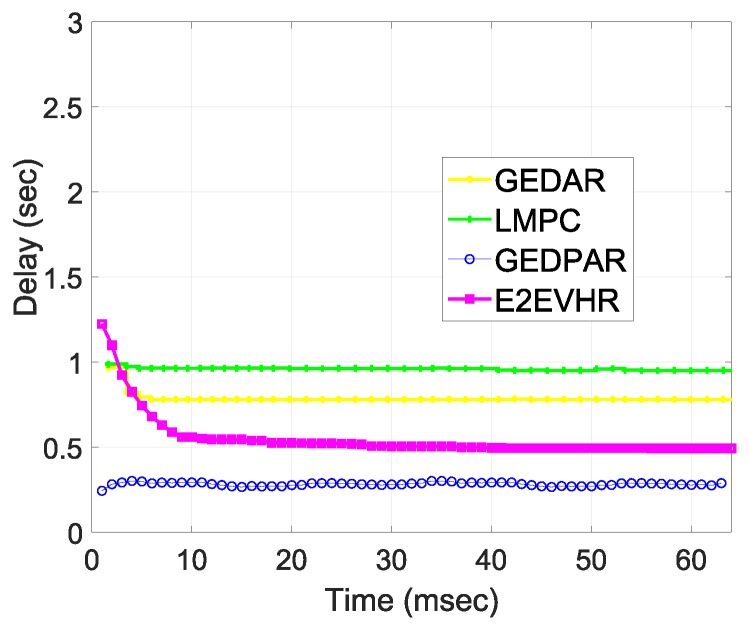
Delay.

Delay is calculated in terms of time duration which is required by a packet to reach from source to destination node. Basically, calculation of delay starts from the time when the first packet is transmitted from the source node. The calculation of delay continues until the last data packet is received at a sink. In addition, delay contains total duration from the generation of the first packet at a source node to the reception of the last packet at the sink node. E2EVHR takes more time at start due to the necessary calculations for source to sink path finding procedure. When it finds the complete path by ensuring that there is no void node in the route then it transfers the packets direclty from selecting paths. On the other hand, GEDPAR involves more calculations and the reason is that it takes more time to the comparing schemes. Delay of the comparing schemes is presented in Figure 13. Moreover, Figure 13 also shows the trade-off and this trade-off authenticates the simulation results. This means that for getting minimum energy consumption and throughput, we have to compromise on computational time.

**Figure 14:**
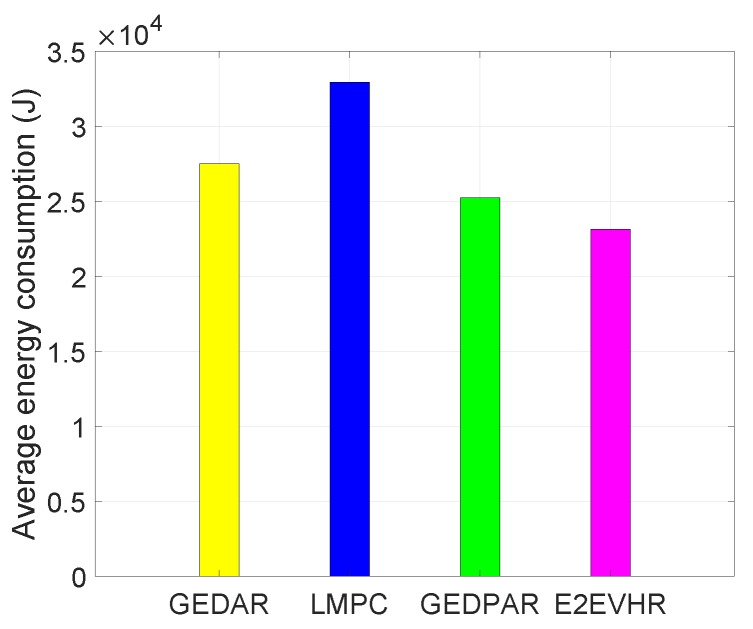
Average energy consumption.

In Figure 14, the comparison among the different routing protocols with respect to energy consumption is presented. From the Figure 14, GEDPAR consumes less energy as compared to the GEDAR and LMPC. GEDAR consumes more energy then counterparts because it focuses on depth adjustment during the void hole avoidance. Depth adjustment takes 1500 mJ energy for one meter while transmission range enhancement takes less energy than depth adjustment (adapted from [22]). Figure 14 is also representing the average energy consumption of all comparing schemes. Simulations are performed multiple times to check the average results. LMPC uses multiple transmissions for one packet which resulted in energy dissipation. The proposed routing protocol (GEDPAR) consumes less energy because it covers the void holes by increasing the transmission range. GEDPAR only changes the depth when no forwarded node is found even by increasing transmission range.

## 6. Feasible Regions

Feasible region is the area where all possible solutions of a particular problem exists. In this section, mathematical formulation using linear programming is used to find the feasibility of the proposed protocol. To achieve the optimal solutions, we define some constraints (listed below). By defining these constraints, the coordinates of feasible regions are calculated. In this work, we have calculated the coordinates for three feasible regions according to the proposed schemes (GEDPAR and E2EVHR).

**Figure 15:**
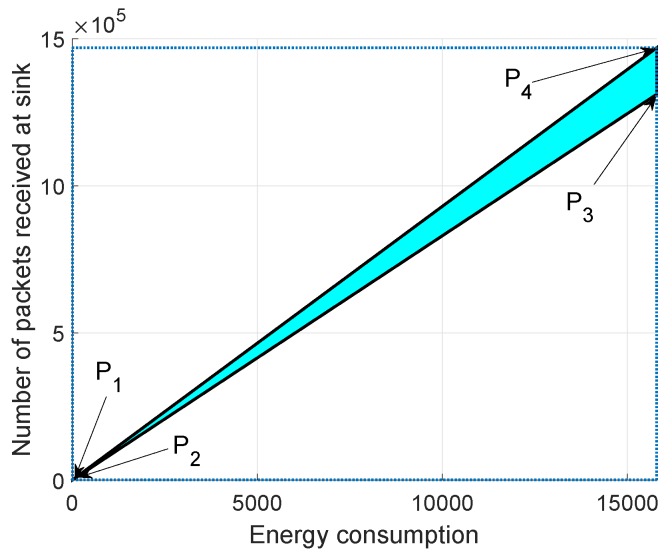
Feasible region for energy consumption and throughput using GEDPAR.

**Figure 16:**
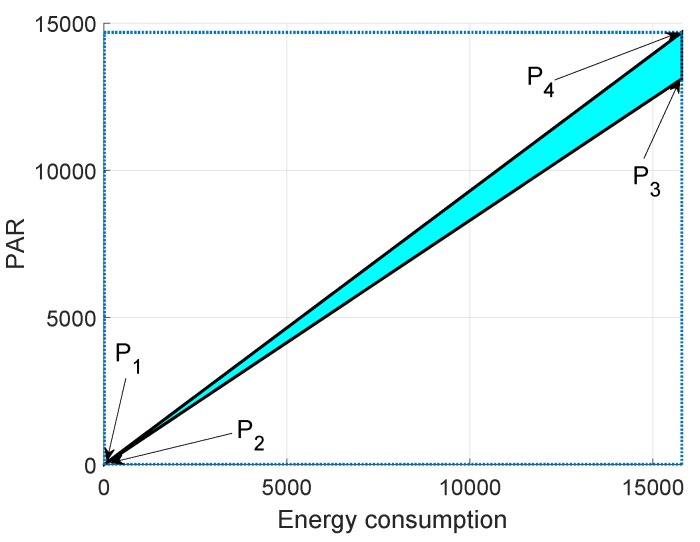
Feasible region for energy consumption and PAR using GEDPAR.

**Figure 17:**
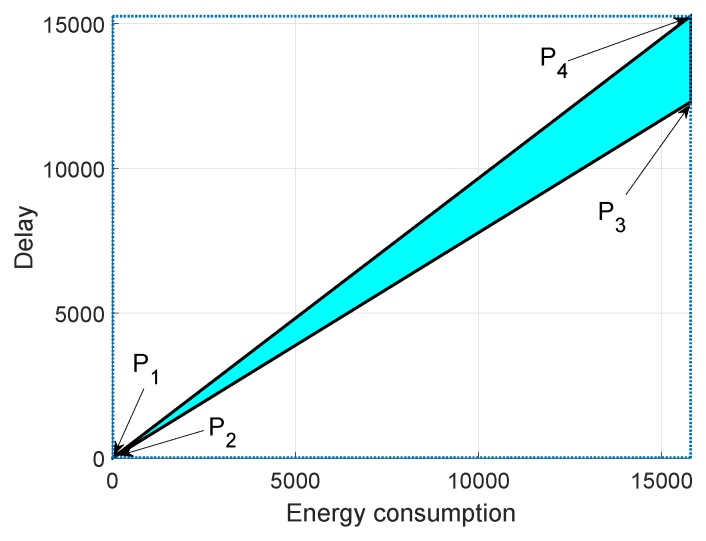
Feasible region for energy consumption and delay using GEDPAR.

In Figure 15, Figure 16 and Figure 17, we annotate the feasible regions as P1, P2, P3 and P4. Figure 15 presents the feasible region of energy consumption versus throughput. Figure 16 illustrates the feasible region (energy consumption versus PAR) for the GEDPAR routing protocol. While in Figure 15, the feasible region of energy consumption versus delay is presented.

**Figure 18:**
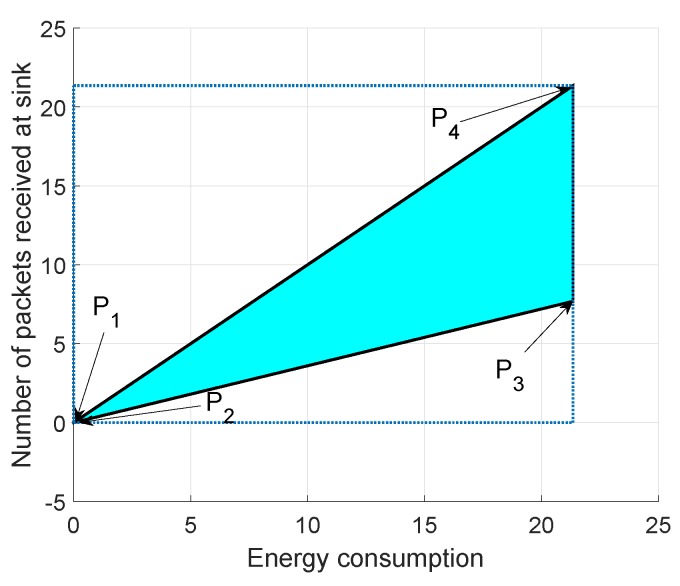
Feasible region for energy consumption and throughput using E2EVHR.

**Figure 19:**
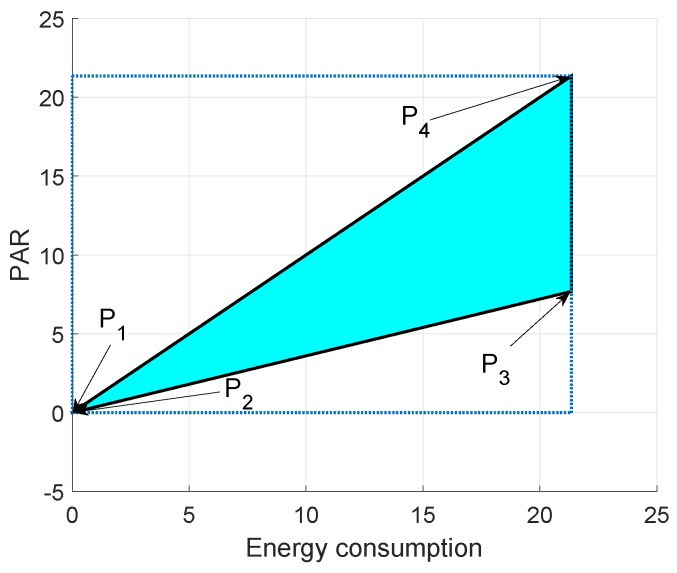
Feasible region for energy consumption and PAR using E2EVHR.

**Figure 20:**
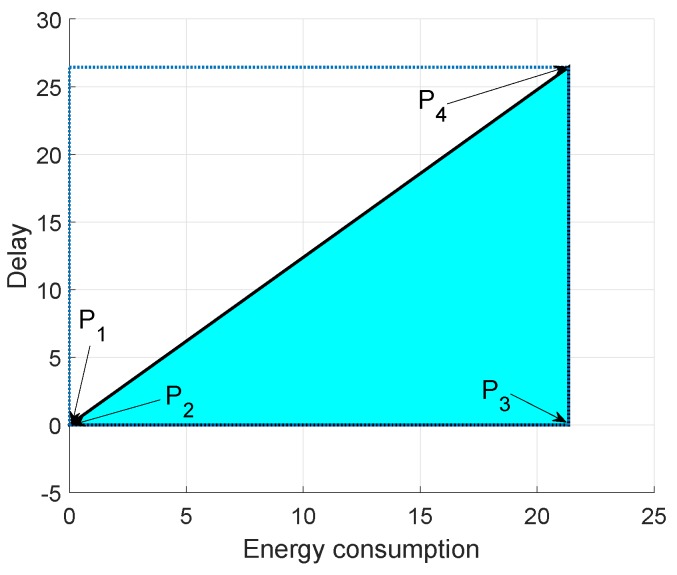
Feasible region for energy consumption and delay using E2EVHR.

In Figure 18, Figure 19 and Figure 20, we annotate the feasible regions for E2EVHR as P1, P2, P3 and P4. Figure 18 presents the feasible region of energy consumption versus throughput, whereas the Figure 19 illustrates the feasible region of energy consumption versus PAR, for the E2EVHR routing protocol. In Figure 18, the feasible region of energy consumption versus delay is presented.

### 6.1. Feasible Region between Energy Consumption and Throughput Using GEDPAR

The feasible region between energy consumption and throughput of the network is calculated by taking the following parameters into account.

- Maximum energy consumption and maximum throughput
- Maximum energy consumption and minimum throughput
- Minimum energy consumption and maximum throughput
- Minimum energy consumption and minimum throughput

Four points are taken in order to draw the feasible region between energy consumption and throughput. Figure 15 shows the feasible region for energy consumption and throughput.

### 6.2. Feasible Region between Energy Consumption and PAR Using GEDPAR

Feasible region for energy consumption and PAR is shown in Figure 16. The four points for the feasible region of energy consumption and PAR are taken as:

- Maximum energy consumption and maximum PAR
- Maximum energy consumption and minimum PAR
- Minimum energy consumption and maximum PAR
- Minimum energy consumption and minimum PAR

### 6.3. Feasible Region between Energy Consumption and Delay Using GEDPAR

Figure 17 depicts the coordinates of feasible region for energy consumption and delay. We take four points between energy consumption and delay to draw the feasible region.

- Maximum energy consumption and maximum delay
- Maximum energy consumption and minimum delay
- Minimum energy consumption and maximum delay
- Minimum energy consumption and minimum delay

### 6.4. Feasible Region between Energy Consumption and Throughput Using E2EVHR

The feasible region between energy consumption and throughput of the network is calculated by taking the following parameters into account.

- Maximum energy consumption and maximum throughput
- Maximum energy consumption and minimum throughput
- Minimum energy consumption and maximum throughput
- Minimum energy consumption and minimum throughput

Four points are taken in order to draw the feasible region between energy consumption and throughput. Figure 18 shows the feasible region for energy consumption and throughput.

### 6.5. Feasible Region between Energy Consumption and PAR Using E2EVHR

Feasible region for energy consumption and PAR is shown in Figure 19. The four points for the feasible region of energy consumption and PAR are taken as:

- Maximum energy consumption and maximum PAR
- Maximum energy consumption and minimum PAR
- Minimum energy consumption and maximum PAR
- Minimum energy consumption and minimum PAR

### 6.6. Feasible Region between Energy Consumption and Delay Using E2EVHR

Figure 20 depicts the feasible region for energy consumption and delay. We take four points between energy consumption and delay to draw the feasible region.

- Maximum energy consumption and maximum delay
- Maximum energy consumption and minimum delay
- Minimum energy consumption and maximum delay
- Minimum energy consumption and minimum delay

## 7. Conclusions

In current work, imbalance and unnecessary energy dissipation are avoided by covering the void hole in an efficient way. We proposed two routing protocols namely GEDPAR and E2EVHR for void hole recovery. To show the efficiency of the proposed protocols, comparative analysis is performed with the existing state of the art protocols: GEDAR and LMPC. Simulations result show that GEDPAR outperforms GEDAR and LMPC in terms of throughput by the percentage of 13% and 37%. PAR of GEDAR, LMPC, E2EVHR and GEDPAR is 0.6, 0.8, 0.93 and 0.95, respectively. While E2EVHR also outperforms the counterparts in terms of throughput, PAR and energy efficiency. However, the proposed protocol (GEDPAR) is minimizing the energy consumption at the cost of affordable delay.

In the future, we will explore and implement some other routing protocols to avoid void holes. To implement these techniques on a test bed for getting more precision in results will be the new direction of our research. An idea about the implementation of “Internet of things” is also under consideration.

## Figures and Tables

**Table 1:** Basic differences between UWSN and WSN.

Base of Difference	UWSN	WSN
Energy consumption	Very high	Low
Propagation delay	High	Low
Bandwidth	Low	High
Dynamic topology operation	High	Low
Efficiency	Low	High
Data transmission rate	Low	High
Environmental and noise interference	High	Low
Communication medium	Acoustic waves	RF waves
Speed of propagation	1200 m/s to 1500 m/s	3 $\times 10^{8}$ m/s

**Table 2:** Comparison of different works.

Type of Technique	Technique/Protocol	Reliability	Packet Size Management	Mobility Management	Number of Hops	Achievements	Challenges	Limitations
**Energy efficiency-based**	RE-PBR [6]	✓	×	✓	Multi-hop	PDR and energy efficiency is enhanced	Difficult to deploy dense network	In dense deployment, end-to-end delay is increased
	TCEB [7]	×	×	×	Multi-hop	Less energy dissipation	Serious cause of energy consumption is attenuation in UWSNs	End-to-end delay is enhanced
	EBLE [8]	✓	✓	×	Single-hop	Lower energy dissipation by balancing the traffic load	Path loss/dead due to continuous data packet transmission	Energy consumption is decreased on the cost of delay
	Cooperative routing [9]	✓	×	✓	Single-hop	Successful packet delivery and lower energy usage	Wireless sensor nodes move with current	Network performance degrades in sparse conditions
	CS [10]	✓	×	×	Single-hop and multi-hop	Beneficial for large amount of data packets	Fewer resources and energy efficiency	Does not perform effectively in Sparse network deployment
	SDVF [11]	✓	✓	✓	Single-hop and multi-hop	Increase network lifetime and PDR	Energy efficiency, network complexity and routing security	Increase in source to destination delay
	MLRP [12]	✓	×	×	Multi-hop	Find efficient path and minimize energy dissipation	Loss of data during transmission process	More memory at each node is required for extra operations
	EBULC [13]	×	×	✓	Multi-hop	Energy efficiency	Complexity of UWSNs	Energy usage is minimizes on the cost of end-to-end delay
	Energy efficient data collecting method [14]	×	×	✓	Multi-hop	Energy efficiency enhanced successfully	Overhead of routing information and increase in operational time	Delay is increased
	Review of existing techniques [15]	✓	×	✓	Single-hop and multi-hop	-	Security issues and energy consumption	Does not discuss the complexity of the reviewed schemes
	Retransmission and redundant approach [16]	✓	×	×	Single-hop	Enhanced PDR	Complexity of the network	Proposed scheme is too much complex to implement
	Integer-linear programming [17]	✓	✓	×	Single-hop	Lifetime of network is increased	Optimal solution for energy dissipation and data packet size	Source to destination delay is increased
**Localization based**	Review on localization algorithms [1]	✓	×	×	Single-hop and multi-hop	-	Malicious attacks	Cannot explain how flooding and path loss problems can be compromised
	Review on localization-based routing algorithms [2]	✓	×	×	Single-hop and multi-hop	-	High interference, limited battery of nodes and low bandwidth	Do not discuss the PDR and void holes
	Review of different techniques [4]	✓	×	×	Single-hop and multi-hop	-	Limited bandwidth, delay problems, localization and security issues	Considerable number of challenges are ignored
	RBCN [18]	×	×	×	Multi-hop	Increase in PDR	Find the locations of alive nodes	End-to-end delay is compromised
	Overview of UWSN works [19]	×	×	✓	Single-hop and multi-hop	-	Localization, hardwares, simulation tools and low-power glider	Issues related to localization are not discussed, e.g., malicious attack
	EEL [5]	×	×	✓	Multi-hop	New algorithm and Improvement in the results	High cost and complexity issues	Cannot find the optimal point for localization and energy usage
**Topology control-based**	TCEB [7]	×	×	×	Multi-hop	Energy consumption is reduced due to dynamic topology	Topology change is not much efficient due to attenuation	End-to-end delay is increased
	Classify topology control algorithm [3]	✓	×	✓	-	-	Mobility of sensor nodes makes difficulty in efficient usage of batteries, loss of connectivity and high bit rate error	Does not provide efficient algorithm
	GARM [20]	×	×	×	Single-hop	PDR and energy efficiency enhanced	Optimal location of glider and minimum channel attenuation	Proposed scheme works better in predefined environment
**Void node-based**	TORA [21]	✓	×	×	Multi-hop	End-to-end delay and alleviation of void holes	Low bandwidth, high latency and error rate	Proposed scheme takes more time on computations
	GEDAR [22]	✓	×	✓	Multi-hop	Void hole avoidance	Computations and energy consumption	Energy consumption for depth adjustment is high
	LMPC [23]	✓	×	×	Multi-hop	Void hole alleviations	Dividing the network area into layers	Communication overhead due to multiple copies, which results in communication delay

**Table 3:** Network parameters setting.

Parameter	Value
Network dimensions	1500 m × 1500 m × 1500 m
Number of sink nodes	45
Other nodes	100
Minimum transmission range	245 m
Maximum transmission range	270 m
Initial energy of nodes	100 J
Velocity of acoustic waves	1500 m/s
Bandwidth	3000 kHz
Packet transmission energy	2 W
Packet reception energy	0.1 W
Idle time energy	10 × $10^{-3}$ W

## Abbreviations

UWSNs	Underwater Wireless Sensor Networks
RF	Radio Frequency
WSN	Wireless Sensor Network
PDR	Packet Delivery Ratio
GPS	Global Positioning System
GEDAR	GEographic and opportunistic based Depth Adjustment Routing
GEDPAR	GEographic and opportunistic based Depth and Power Adjustment Routing
E2EVHR	End to End Void Hole Recovery
LMPC	Layered Multi-path Power Control
PAR	Packet Acceptance Ratio
